# Nanoemulsified orange essential oil for decontamination of some foodborne pathogens on cherry tomatoes during simulated domestic washing

**DOI:** 10.3389/fmicb.2026.1931295

**Published:** 2026-09-07

**Authors:** Ioana M. Bodea, Ana Belén Baenas-Soto, Pilar Truchado, Arturo Esnoz, Alberto Garre, Alfredo Palop

**Affiliations:** 1 Universidad Politécnica de Cartagena Member of European University of Technology EUT+, Departamento de Ingeniería Agronómica, Instituto de Biotecnología Vegetal, Cartagena, Spain; 2 Research Group on Microbiology and Quality of Fruit and Vegetables, CEBAS–CSIC, Murcia, Spain

**Keywords:** decontamination, food safety, natural antimicrobials, orange essential oil, risk assessment

## Abstract

Surface decontamination is key for ensuring the food safety of ready-to-eat (RTE) produce. However, current chlorine-based washes raise health concerns, highlighting the need for alternative decontamination strategies, in response to increasing regulatory restrictions across several European countries. Thus, the aim of this study was to assess the potential of an alternative solution based on nanoemulsified orange essential oil (OEO) for the disinfection of relevant foodborne pathogens like *Escherichia coli* O157:H7 (*E. coli*), *Salmonella enterica* subsp. enterica ser. Enteritidis (*S.* Enteritidis) and *Listeria monocytogenes* (*L. monocytogenes*). The antimicrobial effect of different OEO concentrations (0.33, 1.66, 5, and 10%) and exposure times (2, 5, 10, and 15 min) were evaluated, using water washing as baseline (no antimicrobial effect) and washing with a commercial chlorine solution as a reference. Both OEO concentration and the contact time increased the antimicrobial effect, reaching a plateau at high value. The highest concentration (10%), applied for 5 min, achieved reductions of up to 2 log CFU/g for *E. coli* and *S*. Enteritidis, comparable to the commercial chlorine-based wash, but only ∼1 log CFU/g against *L. monocytogenes*. Extending the washing time to 15 min further increased reductions to 2.5 and 3.5 log CFU/g for *E. coli* and *S.* Enteritidis, respectively, demonstrating the effectiveness of OEO under simulated domestic washing conditions. Based on a quantitative microbial risk assessment (QMRA), the use of the alternative solution was estimated to maintain consumer protection against salmonellosis and shiga-toxin producing *E. coli*, highlighting its potential as a chlorine-free washing treatment for fresh produce.

## 1 Introduction

The demand for fresh fruits and vegetables has increased worldwide as key components of healthy and balanced diets (Anand et al., 2008; Boeing et al., 2012). Tomatoes are one of the main fruits produced worldwide, with FAO estimating a global production in 2023 of 192 million tons, China being the main producer (FAOSTAT, 2025). They are of high interest for healthy diets due to their high content in vitamin C, potassium, and fibers, as well as the antioxidant lycopene (Beecher, 1998).

On the other hand, fresh produce also presents a potential consumer hazard, as foodborne pathogens may contaminate the product (Santos et al., 2022; Melo and Quintas, 2023). *Salmonella* outbreaks associated with tomatoes are relatively common (EFSA, 2023, 2024), such as the *Salmonella* Strahcona outbreak that resulted in 427 confirmed cases across 17 Member States between 2023 and 2025 (EFSA, 2024). *E. coli* is another important pathogen that may be present in fresh produce (Harvey et al., 2016), has been isolated from tomatoes (Gomez-Aldapa et al., 2013) and has been linked to several recalls (EFSA, 2011; CDC, 2024). *L. monocytogenes* is also a main concern for RTE foods (EFSA et al., 2020; CDC, 2023). Although it is not often isolated from tomatoes, this pathogen may persist in food production environments (Gil et al., 2024; Markovich et al., 2025), potentially contaminating tomato surfaces during processing, where it can attach and survive (Cabrera-Diaz et al., 2022). Therefore, these microorganisms are often selected as relevant foodborne pathogens associated with fresh produce contamination, including cherry tomatoes. *S.* Enteritidis (EFSA, 2024; ECDC, 2025) and *E. coli* (Burger, 2012; CDC, 2024) represent common fresh produce associated pathogens. *L. monocytogenes* represents a main concern for RTE products, due to its relatively high hospitalization (>95%) and mortality (>15%) rates (EFSA et al., 2020; European Food Safety Authority [EFSA] and European Centre for Disease Prevention and Control [ECDC], 2025), as well as its ability to thrive at refrigeration temperatures on acidic media. Therefore, it is a pathogen of relevance for fresh tomatoes.

The control of fresh produce can be challenging because their fresh nature limits the application of thermal treatments, as they might impact their nutritional and sensory properties (Possas et al., 2021; Santos et al., 2022). Instead, bacterial contamination in tomatoes is typically managed by washing using water or chemical sanitizers such as chlorine or sodium hypochlorite (Santos et al., 2022). While these sanitizers are widely recognized as effective and safe when used at approved concentrations, their application is subject to increasing regulatory scrutiny, and concerns have been raised regarding exposure, and the potential formation of by-products under certain conditions (Banach et al., 2021; Bodea et al., 2023). Accordingly, several changes in legislation have restricted their use in some European countries, namely Belgium, the Netherlands, Sweden, Germany, Switzerland, and Denmark (Santos et al., 2022; Luciano et al., 2025), whereas other countries, such as Spain have reduced their maximum concentration to 80 ppm (Ministerio de la Presidencia, Justicia y Relaciones con las cortes, 2023). Therefore, there is a need for the development of novel washing solutions that use alternative decontaminating agents.

Essential oils (EOs) are natural plant products with antimicrobial properties, which are increasingly recognized as promising antimicrobial compounds against bacteria and yeasts (Swamy et al., 2016). They have a Generally Recognized As Safe (GRAS) status by the Food and Drug Administration (FDA), which makes them of high interest for the produce industry as alternative disinfectants (Luciano et al., 2025). However, their low water solubility, low stability and strong aromatic properties present notable challenges for their application by the food industry (Nieto et al., 2023).

Previous research has highlighted that the encapsulation of EOs in nanoemulsions can overcome some of these challenges, by improving their dispersion, stability, and antimicrobial performance, while enabling the use of lower EO concentrations. Therefore, nanoemulsification represents a promising strategy for incorporating EOs into aqueous washing treatments (Prakash et al., 2018; Buendía-Moreno et al., 2019). Nanoemulsification enhances EO performance by improving dispersion and stability in aqueous systems, while increasing the surface area available for interaction with bacterial cells, potentially improving antimicrobial efficacy (Guerra-Rosas et al., 2017). Additionally, previous studies have assessed the potential of nanoemulsified EO based washing solutions to act as alternatives to current chlorine-based compounds (Guerra-Rosas et al., 2017; Dávila-Rodríguez et al., 2019; Gurtler et al., 2022; Santos et al., 2022; Bodea et al., 2023). These studies reported concentration-dependent antimicrobial effects of EO nanoemulsions. Bodea et al. (2023) observed increased *E. coli* reductions with higher concentrations of oregano and rosemary EO nanoemulsions, while Luciano et al. (2025) reported similar trends for limonene. Guerra-Rosas et al. (2017) showed that bacterial susceptibility varies among species, with *E. coli* being more sensitive than *Listeria innocua* to mandarin EO nanoemulsions. However, this technology is still emerging and not yet fully established, requiring further research to address existing knowledge gaps, including the identification of the most effective EO against each foodborne pathogen, their optimal application method and potential interaction with the food matrix. In particular, while orange EOs (OEO) has already showed antibacterial activity against foodborne pathogens *in vitro*, (Calo et al., 2015; Angane et al., 2022), by minimum inhibitory concentration (MIC), bactericidal concentration (MBC) or disk diffusion test, limited information is available on its effectiveness as a nanoemulsified washing solution for fresh produce. Furthermore, OEO was selected not only for its antimicrobial potential but also for its regional availability and sustainability. The Murcia region (Spain) is one of the main producers and exporters of oranges, generating large quantities of peel byproducts that remain underutilized. Therefore, valorizing these residues as a source of OEO could represent a more economically and environmentally sustainable alternative, even if its antimicrobial activity is lower than that of other EOs, such as oregano or thyme EOs.

Therefore, this study aims to fill that knowledge gap, and provides a proof-of-concept evaluation on the decontamination effect of washing solutions based on nanoemulsified OEO, extracted from orange peel waste, as an effective alternative to conventional chlorine-based solutions. Since domestic consumers apply washing solutions directly to fresh produce, to reduce surface contamination, the effectiveness of the nanoemulsified OEO was evaluated under conditions representative of household use. Experiments were conducted on artificially contaminated cherry tomatoes inoculated with *E. coli, S.* Enteritidis, or *L. monocytogenes*, and compared with water washing (a baseline without disinfectant effect) and chlorine washing (a target disinfection level).

Although bacterial reductions are important indicators of decontamination efficacy, they do not directly reflect the potential impact on consumer health. Quantitative microbiological risk assessment (QMRA) provides a flexible framework to translate pathogen reductions into estimates of changes in foodborne illness risk (WHO, 2021). After hazard identification, this framework combines predictions of bacterial reductions with other mathematical models to describe changes in microbial concentration throughout the food supply chain to estimate consumer dose of microbial hazards (exposure assessment). These estimates can be combined with dose-response models (hazard characterization), facilitating the evaluation of alternative washing strategies and their potential impact on food safety under different scenarios (risk characterization). In our current study, we built mathematical models to perform a quantitative approach for risk assessment, as it provides a more detailed description of risk than qualitative approaches (Coleman and Marks, 1999).

## 2 Materials and methods

### 2.1 Extraction of orange essential oil

OEO was extracted by ultrasound assisted hydro-distillation (UAHD) from orange peel waste, as previously described by Bodea et al. (2026). The orange waste (peel and pulp) from Navelina, Navel, and Navel Late orange (*Citrus sinensis* L. Osbeck) was collected after juicing, and the pulp was manually removed from the peel. The obtained peels were grinded into small pieces (1–5 mm) with a household blender (Cecotec Power Titanium Pro 2000 W 32500 rpm), for 10 s at highest speed setting (maximum power of 32,500 rpm), portioned into 50 g samples and freezed until further use.

Before the extraction, one sample (50 g) was defrosted at room temperature (approx. 30 min), and added to 250 mL of distillated water. The obtained mixture was then submerged to UAHD. Ultrasound treatment was carried out with a Hielscher UP400St sonicator (Hielscher Ultrasonics, Germany) equipped with a S24d7 sonotrode tip working at a constant power of 400 W and a frequency of 50-60 Hz. The working period was set continuously for 10 min, at 100% amplitude according to previous results (Bodea et al., 2026). The ultrasound treatments were applied at room temperature, and the resulting treated samples were submitted to conventional hydro distillation (HD), using a Clevenger apparatus (Vidra FOC, 519 A/1, Spain), for 3 h. The obtained EO was collected and dried with anhydrous sodium sulfate and kept at 4 °C in amber vials until further use. The UAHD extraction method was previously compared with conventional HD in terms of extraction yield, extraction rate, EO composition, and energy consumption, and detailed results were previously reported in our earlier study (Bodea et al., 2026), to which the reader is referred for further information.

### 2.2 Preparation and characterization of nanoemulsions

OEO nanoemulsions were prepared following the method described by Bodea et al. (2023) with several modifications. First, distilled water (3.55 mL) and propylene glycol (2.75 mL) (Panreac, Barcelona, Spain) were mixed to obtain the aqueous phase. Afterwards, 2.00 mL of OEO were mixed with 1.20 mL of Tween 80 (Panreac) and 0.50 mL sunflower oil (Hacendado, Valencia, Spain), to obtain the oily phase. The emulsions were then prepared by combining the two phases, obtaining a final volume of 10 mL. Subsequently, the emulsions were subjected to sonication using a Hielscher UP400St ultrasonic processor (Hielscher Ultrasonics) equipped with an S24d7 sonotrode tip working at a constant amplitude of 100% and a power of 400 W. The working period was set to 30 s with a pause of 5 s between working times until the imposed energy limit of 500 Ws was reached. The ultrasonic treatment was applied 5 consecutive times, with the same input settings. The temperature was kept constant (∼20°C) by placing the emulsion tube in an ice bath. Fresh nanoemulsions were prepared independently on the day of each experiment, and used immediately after preparation.

The droplet size distribution of the oil particles was determined by laser light scattering method using a Mastersizer 2000 (Malvern Instruments, Worcestershire, United Kingdom). OEO nanoemulsions had a median droplet size [D (0.5)] of 0.201 μm, and a Sauter mean diameter [D (3,2)] of 0.218 μm, which is similar to previously reported results (Jaiswal et al., 2015; Bodea et al., 2023). The stability of the OEO nanoemulsions was evaluated by monitoring droplet size during refrigerated storage. No significant changes were observed during 1 month of weekly measurements, however, droplet size increased thereafter, indicating the onset of destabilization.

### 2.3 Microorganisms and culture conditions

*E. coli* O157:H7 CECT 5947, *Salmonella enterica* subsp. *enterica* ser. Enteritidis CECT 4155 and *L. monocytogenes* CECT 4032 were provided by the Spanish Type Culture Collection (Valencia, Spain) and stored at −80°C in cryovials until use. A loop of bacteria cells from each strain was individually transferred on Luria-Bertani agar (LB; Scharlau Chemie S.A., Barcelona, Spain) for both *E. coli* and *S.* Enteritidis, while for *L. monocytogenes* tryptic soy agar was used (TSA; Scharlab Chemie S.A.). All bacteria strains were grown overnight at 37°C on their specific agar media. A single colony from each bacteria strain was individually inoculated in 5 mL sterile LB broth (Scharlab Chemie S.A.), for *E. coli* and *S.* Enteritidis, and tryptic soy broth (TSB; Scharlab Chemie S.A.) for *L. monocytogenes* and incubated for 24 h at 37 °C. Afterwards, 1 mL of each inoculum was sub-cultured in 500 mL specific broth media, and incubated at 37°C for 24 h under agitation to obtain bacteria in the early stationary growth phase, with an initial bacterial count of approximately 10^7^ CFU/mL, which were subsequently used for tomato inoculation. The inoculum concentration was verified by serial dilution and plating on the corresponding agar medium, for each bacteria strain.

### 2.4 Inoculation of cherry tomatoes

Cherry tomatoes were purchased from a local market (Cartagena, Spain). Batches with uniform shape, color, and mass (∼10 g each) were selected for the experiments, and kept at 4 °C until treatment. Before the decontamination treatments, the cherry tomatoes were washed with potable tap water to simulate domestic behavior. The tomatoes were placed in a metal sieve and rinsed under a fresh flowing stream of cold water (20 °C) for 1 min until covering the full surface, to eliminate residues, and then dried in a Class II biological safety cabinet (Telstar Technologies BIO II A, Spain) under aseptic conditions, for 1 h. Afterwards, the cherry tomatoes were artificially contaminated with *E. coli*, *S.* Enteritidis or *L. monocytogenes* by dipping 3 tomatoes/sample (∼30 g) in the 500 mL of overnight culture of every strain, for 30 min, to allow the attachment of bacteria on the surface. Subsequently the inoculated tomatoes were left to dry on a sterile metal rack, in a biological safety cabinet under aseptic conditions, for 1 h, with rotation after 30 min to ensure uniform drying and bacterial attachment on the surface. The tomatoes were placed in a metal sieve and rinsed under a fresh flowing stream of cold water (20 °C) for 1 min, ensuring complete surface coverage to remove residues, and were then dried for 1 h in a Class II biological safety cabinet under aseptic conditions.

### 2.5 The decontamination effect of washing with distilled water or chlorine solutions

The antimicrobial efficacy of distilled water and two different concentrations of chlorine solutions in distilled water was assessed individually against *E. coli*, *S.* Enteritidis and *L. monocytogenes*, as previously described by Bodea et al. (2023). Three artificially contaminated cherry tomatoes (∼30 g) were used for each experimental repetition. Fresh washing solution was prepared and used for each replicate to ensure consistent treatment conditions and avoid potential changes in antimicrobial activity associated with repeated use. The previously contaminated tomatoes were immersed in 300 mL of each treatment solution for 5 min without agitation, to assess the effect of the washing treatments. The 5 min treatment duration was selected as it aligns with the time recommended by the commercial chlorine solution provider (Sanicentro, Guadalajara, Spain). Two chlorinated solutions were prepared: one according to the manufacturer’s instructions, 2.5 mL of 10 g/L sodium hypochlorite were added to 297.5 mL distilled water, resulting a final concentration of 80 ppm active chlorine; and the second chlorine solution had a final concentration of 160 ppm (double of the previous one). The two chlorine concentrations were selected as 80 ppm free chlorine aligns with the limits established by the new legislation in Spain (Ministerio de la Presidencia, Justicia y Relaciones con las cortes, 2023), while the second is twice that value, reflecting concentrations used under the previous regulatory framework, for which more comparative data is available. Additionally, the initial bacterial concentration, of each strain, on the artificially contaminated tomatoes was determined for each experimental condition and resulted in a bacterial concentration of ∼7 log CFU/g on the recovery media. The determinations for each condition were performed in triplicate, for each bacterial strain.

### 2.6 The decontamination effect of washing with nanoemulsified OEO

The effect of the different washing treatments with concentrations of 0.33, 1.66, 5, and 10% OEO nanoemulsion in distilled water were assessed against *E. coli*, *S.* Enteritidis and *L. monocytogenes*. The selected OEO concentrations were based on our previous work (Bodea et al., 2023) and preliminary experiments, which showed that higher concentrations did not result in additional antimicrobial efficacy against any of the three tested bacteria. Distilled water and an 80 ppm chlorine solution were used as negative and positive controls, respectively, to provide baseline and reference antimicrobial performance. These treatments were designed to reflect domestic washing practices, where different concentrations of disinfectant solutions may be applied depending on consumer preferences. The cherry tomatoes were prepared as previously described. Briefly, cherry tomatoes were contaminated for 30 min with each bacteria strain. Subsequently, 3 artificially contaminated cherry tomatoes (∼30 g) were immersed in 300 mL of each washing solution for 5 min without agitation. A positive control, without any washing treatment, was performed for each experiment to assess the initial contamination on cherry tomatoes and the effectiveness of the washing treatments. The initial bacterial concentration on the artificially contaminated tomatoes, was determined for each experimental condition, resulting in ∼7 log CFU/g (10^7^ CFU/g), whereas the native microbial load of fresh tomatoes was measured at < 2 log CFU/g (validated by plate count on controls). Each condition was assessed in triplicate, for all three bacterial strains.

### 2.7 The impact of treatment time on effectiveness of the nanoemulsified OEO

After assessing the effectiveness of the treatment and identifying the most effective concentration (10%), further experiments were conducted. Preliminary tests showed that the solution volume did not impact the results (data not shown), so these experiments used a volume of 100 mL. The effect of the treatment time on bacterial reduction was performed using a contact time of 2, 5, 10, and 15 min, and was assessed against *E. coli* and *S.* Enteritidis. As the OEO nanoemulsion was ineffective against *L. monocytogenes* even at higher tested concentration (up to 20%), this pathogen was excluded from further experiments. The selected contact times (2–15 min) were chosen to represent the range of immersion times previously evaluated for household washing treatments, as studies assessing washing treatments reported immersion times ranging from 1 to 60 min (Bhilwadikar et al., 2019) and indicated that exposure periods longer than 10 min in household applications provided limited additional antimicrobial benefits (Nastou et al., 2012). Additionally, an 80 ppm chlorine solution, used as baseline, was applied for 5 min, as recommended by the manufacturer.

A lower bacterial load was used to better reflect realistic contamination levels expected under consumer conditions, as the initially applied levels were unlikely to occur in real-life scenarios. This adjustment enhances the practical applicability of the results. The cherry tomatoes were contaminated for 30 min with *E. coli* or *S.* Enteritidis. The contamination solution was prepared by inoculating a single colony from each bacteria strain in 5 mL sterile LB broth, and incubated for 24 h at 37 °C. Afterwards, the 5 mL inoculum was diluted in 495 mL of peptone water to reduce the initial microbial load (2 log reduction), resulting in a final concentration of approximately 10^5^ CFU/mL, and a bacterial concentration of ∼5.5 log CFU/g on the recovery media. The determinations for each condition were performed in triplicate, for each bacterial strain.

### 2.8 Bacteria enumeration

Treated cherry tomatoes (∼30 g), were homogenized in 270 mL peptone water for 1 min using a masticator (Colwort Stomacher 400 Lab, Seward Medical, London, United Kingdom). Afterwards, serial decimal dilutions in peptone water were performed, and 1 mL of the appropriate dilution was plated in specific recovery media (LB for *E. coli* and *S.* Enteritidis, and TSA for *L. monocytogenes*). The plates were incubated at 37°C for 24 h and the results were expressed in colony-forming units per gram (CFU/g) to determine the microbial load, or log-transformed (log CFU/g) and expressed as the number of log reductions. This was determined for each batch by subtracting the bacterial counts (log CFU/g) after washing from the initial counts before treatment. The detection limit of the bacterial enumeration method was 30 CFU/g (1.5 log CFU/g). The enumeration method was also performed for uninoculated tomatoes to ensure the lack of bacteria on the produce before the artificial contamination. All experiments were performed in triplicate.

### 2.9 Quantitative microbiological risk assessment for *Salmonella* Enteritidis on cherry tomatoes

#### 2.9.1 Contamination pathway

The QMRA model was implemented as a modular process risk model (MPRM) (Nauta, 2007), a methodology typically used to evaluate consumer exposure and risk assessment for foodborne bacteria (WHO, 2021). The goal of the assessment is to compare an alternative scenario where the OEO washing solution is used for tomato decontamination against current solutions in Western countries. Hence, the contamination pathway considered was tomatoes contaminated by *S.* Enteritidis on surface due to cross-contamination at field level due to water splashing. The microbial population would survive washing and grow during storage until consumption. *S.* Enteritidis was selected because it is more relevant for tomato than *L. monocytogenes* or *E. coli* (EFSA, 2013).

Table 1 summarizes the parameters and probability distributions for the QMRA. The calculations considered three scenarios:

- Worst-case scenario: water washing
- Best-case scenario: washing with a chlorine solution at 160 ppm (no longer legal in Spain)
- Alternative scenario I: washing with a chlorine solution at 80 ppm
- Alternative scenario II: washing with a solution based on 10% nanoemulsified OEO.

**TABLE 1:** Parameter estimates and Root Mean Squared Error (*RMSE*) for the predictive models fitted to describe the effect of the OEO concentration or the washing time on the antimicrobial effect of the alternative washing solution.

Parameter	Estimate ± standard error
*a* (log CFU/g)	1.5 ± 0.6
*b* (log CFU/g)	2.6 ± 0.6
*log*⁡*d* (log min)	0.65 ± 0.08
*RMSE* (log CFU/g)	0.18

#### 2.9.2 Prevalence an initial concentration

Prevalence of *S.* Enteritidis in tomatoes was set at 2%, based on an empirical study performed on the United States (Reddy et al., 2016). The logarithm of the microbial concentration in those samples was defined as a uniform distribution with limits log_10_(3) and log_10_(117) log CFU/g according to the results reported by Cevallos-Cevallos et al. (2012) based on an empirical study on the cross-contamination of tomato surface due to water splashing.

#### 2.9.3 Reduction in *S.* Enteritidis concentration after washing

The reduction in *S.* Enteritidis concentration after different types of washing was estimated based on the empirical results obtained in this study. For water washing and commercial chlorine washing, a normal probability distribution was fitted to the difference between the initial concentration and final concentration observed by maximum likelihood using the *fitdistrplus* R package (Pouillot and Delignette-Muller, 2010), obtaining the results reported in Table 2.

**TABLE 2:** Results of the QMRA model for risk of Salmonella Enteritidis associated to the consumption of cherry tomatoes.

Scenario	Concentration at consumption (log CFU/g)	Number of cases per 1.000.000 servings
Water wash	0.824 (0.08, 1.57)	1.7 ⋅ 10^–3^ (6.4 ⋅ 10^–4^, 2.9 ⋅ 10^–3^)
Chlorine wash (80 ppm)	−0.81 (−1.57, −0.04)	1.3 ⋅ 10^–4^ (0, 6.3 ⋅ 10^–4^)
Chlorine wash (160 ppm)	−0.98 (−1.17, −0.25)	8.7⋅ 10^–5^ (0, 4.5 ⋅ 10^–4^)
Nanoemulsified EO wash (10 %)	−1.25 (−2.54, −0.085)	9.8⋅ 10^–5^ (0, 5.7 ⋅ 10^–4^)

The output is reported as the median of 10 million Monte Carlo simulations, with the 10th and 90th percentiles within brackets.

The antimicrobial effect of the OEO washing solution was impacted by the contact time. Therefore, an empirical equation (Equation 1) was used to describe the impact of the washing time (*t_w_*) on the number of log-reductions (*R*). The empirical equation includes three parameters: *a* (corresponding to the number of log-reductions for *t*_*w*_0), *b* (related to the slope of the *R* vs. *t_w_* relation for low *t_w_*) and *d* (linked to a plateau in the relationship for higher *t_w_*).


$$R=a+b\cdot \frac{t_{w}}{t_{w}+d}$$

The values of the three parameters were estimated by non-linear regression from the number of reductions observed for different washing times using a 10% OEO washing solution. Parameter uncertainty was described using a normal distribution, with mean value the estimated value and standard deviation the standard error of regression.

The uncertainty in the contact time was described as a triangular distribution, with minimum value 3 min, maximum value 6 min and most likely 4 min.

#### 2.9.4 Growth during storage

The growth of *Salmonella* spp. on tomatoes during storage was described using the predictive model proposed by Pan and Schaffner (2010), based on artificially inoculated tomatoes. The model proposed a linear relationship between the storage temperature (*T*) and the square root of the specific growth rate ($\sqrt{\mu }$). Model parameters were fixed to the values reported in the original study, as it did not report parameter uncertainty.

The variability in growth temperature during domestic storage was described based on the on-site measurements reported by Jofré et al. (2019), as a triangular distribution with parameters based on the results reported as most likely for the core of a refrigerator.

#### 2.9.5 Consumer phase and dose-response model

Hazard characterization was based on the dose-response model reported by Pouzou et al. (2025), who reported a beta-Poisson model for *S.* Enteritidis. The serving size was described as a uniform distribution, with limits of 10 and 60 g, with the dose calculated as the concentration at exposure multiplied by the serving size.

### 2.10 Statistical methods and computer implementation

All calculations were implemented in R version 4.2.3 (R Core Team, 2022). The QMRA model was implemented as a Monte Carlo simulation using the *biorisk* package for R (available online at https://github.com/albgarre/biorisk). Calculations were repeated for an increased number of Monte Carlo iterations until quantiles of exposure and number of cases converged, resulting in 10,000,000 iterations. Common statistical methods (ANOVA analysis, Tukey HSD) were also implemented in R version 4.2.3, using a confidence level α0.05.

## 3 Results and discussion

### 3.1 Decontamination effect of conventional treatments (water and chlorine) against *E. coli*, *S. Enteritidis*, and *L. monocytogenes*

**FIGURE 1:**
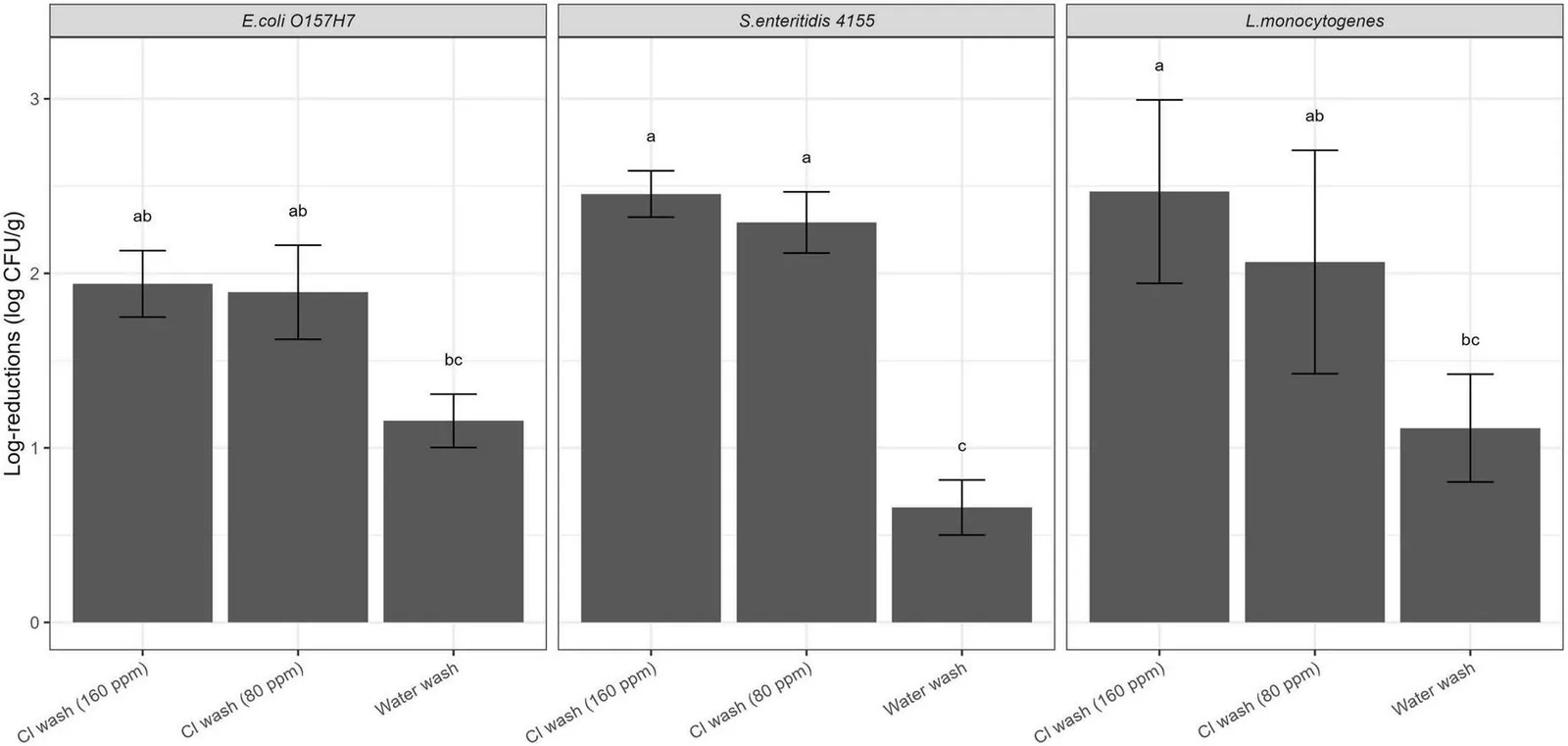
The differences between bacterial concentrations of *E. coli*, *Salmonella* Enteritidis and *Listeria monocytogenes* on artificially contaminated tomatoes after washing with water (Water wash), after washing with a commercial chlorine-based solution at 160 ppm (Cl wash 160 ppm) and washing with a commercial chlorine-based solution at 80 ppm (Cl wash 80 ppm) for 5 min. Letters indicate the output of a *post-hoc* Tukey test (α0.05)

When assessing the effect of washing with water and chlorine on bacterial reductions, results showed that the decontamination potential varied significantly depending on the bacterial species and treatment type (Figure 1). Water washing had a similar effect against all three tested bacteria, displaying an average reduction of 1.10 log CFU/g against *E. coli* and *L. monocytogenes*, while showing a lower reduction of 0.66 ± 0.30 log CFU/g against *S.* Enteritidis. This result is in line with previous studies (Banach et al., 2021) and serves as a baseline representing a scenario where bacterial reduction is attributed to the passive removal of loosely attached microorganisms during water immersion, rather than to antimicrobial activity or additional external factors such as agitation.

No significant differences were observed between the 80 and 160 ppm chlorine treatments for any of the tested microorganisms (Tukey test, α = 0.05), indicating that the higher chlorine concentration did not provide additional reductions under the tested conditions (Figure 1). Compared to water washing, chlorine washing was most effective against *S.* Enteritidis, displaying reductions of 2.29 ± 0.33 and 2.46 ± 0.25 log CFU/g, for chlorine at 80 ppm and 160 ppm, respectively. The decontamination effect against *L. monocytogenes* was comparable, with bacterial reductions of 2.07 ± 1.23 log CFU/g and 2.48 ± 1.00 log CFU/g for both 80 ppm and 160 ppm chlorine solutions. Finally, cherry tomatoes inoculated with *E. coli* showed slightly lower bacterial reductions (1.94 ± 0.37 log CFU/g and 1.89 ± 0.52 log CFU/g for chlorine at 160 ppm and 80 ppm, respectively).

These results are generally consistent with previous studies evaluating chlorine decontamination treatments of tomatoes, although the reported antimicrobial efficacy varies considerably depending on the experimental conditions. Overall, chlorine consistently achieved greater bacterial reductions than water washing, but the magnitude of this effect was influenced by factors such as inoculation method, chlorine concentration, organic matter, and the target microorganism. Bolten et al. (2020) observed that chlorine was significantly more effective than distilled water (*p* < 0.02) against *Salmonella* on tomatoes, although no clear relationship between chlorine concentration (25–150 mg/L) and efficacy was observed. Similarly, Gurtler et al. (2022) reported reductions of approximately 2.5–2.6 log CFU/g for *Salmonella*, *L. monocytogenes*, and *E. coli* O157 on dip-inoculated cherry tomatoes treated with 8 ppm free chlorine, although these reductions were not significantly different from those achieved by water washing (1.7 log CFU/g). In contrast, Chen et al. (2019) observed substantially greater reductions with 200 ppm chlorine than with water, particularly against *L. monocytogenes*. Washing spot-inoculated cherry tomatoes with distilled water for 3 min resulted in reductions of 0.63, 0.71, and 1.36 log CFU/g for *E. coli* O157:H7, *S. enterica*, and *L. monocytogenes*, respectively. In contrast, treatment with a pH 6.5-adjusted 200 ppm chlorine solution achieved significantly greater reductions of 2.47, 1.06, and 5.51 log CFU/g, respectively. Moreover, Gurtler (2020) showed that chlorine efficacy was markedly reduced in the presence of organic matter, with bacterial reductions below 0.76 log CFU/mL under high organic load compared with up to 2.77 log CFU/mL under cleaner conditions. The authors observed that by using a 46 ppm chlorine solution in the presence of 0.3% bovine serum albumin (BSA), *E. coli* O157:H7, *S. enterica* and *L. monocytogenes* were inactivated by less than 0.76 log CFU/mL after a 5 min treatment, while under cleaner conditions (0.03% sterile BSA), lower concentrations of chlorine (9 ppm and 15 ppm) achieved log reductions of 0.64 and 2.77 log CFU/mL, respectively (Gurtler, 2020). Additionally, although the same experimental conditions were applied to all replicates, the higher variability observed for *L. monocytogenes* after chlorine treatment may be associated with the inherent heterogeneity of fresh produce matrices, including differences in bacterial attachment, surface characteristics, and microbial recovery efficiency (Ijabadeniyi et al., 2011). Together, these studies demonstrate that chlorine efficacy depends not only on disinfectant concentration but also on experimental conditions, emphasizing the importance of considering the application scenario when comparing decontamination strategies.

### 3.2 Decontamination effect of nanoemulsified OEO solutions compared to conventional ones

#### 3.2.1 Effect of the OEO concentration on the antimicrobial effect of the alternative wash

The antimicrobial effect of the conventional chlorine solution was used as target for the formulation of the alternative solution based on nanoemulsified OEO. First, the OEO concentration was modified, maintaining an exposure time of 5 min (the same used for the chlorine washing). The decontamination efficacy of the nanoemulsified OEO treatments varied considerably with bacteria species and EO concentration. Overall, OEO nanoemulsions showed reductions comparable to those obtained with the chlorine solution under the tested conditions against *E. coli* and *S*. Enteritidis, whereas limited antimicrobial effectiveness was observed against *L. monocytogenes*.

**FIGURE 2:**
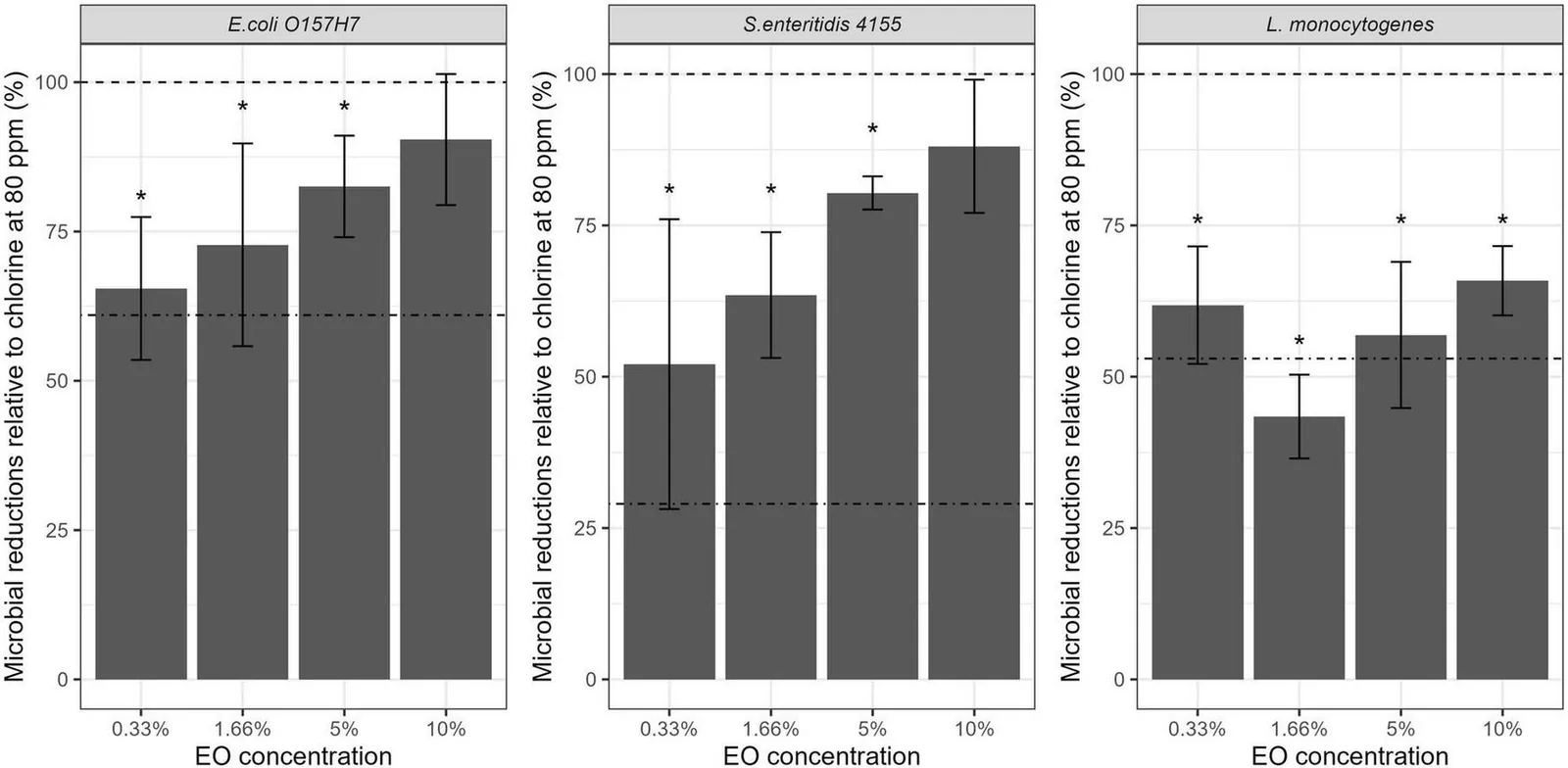
The relative bacterial reductions (% of log-reductions) obtained with nanoemulsified OEO with different concentrations (0.33–10%) against *E. coli* O157:H7, *Salmonella* Enteritidis, and *Listeria monocytogenes* reductions on cherry tomatoes. Values are expressed as percentages relative to the reductions obtained with a chlorine solution at 80 ppm (100%) (–). The dotted line represents the reductions obtained with water washing (.-.). Error bars represent the standard deviation. Asterisks (*) denote significant differences compared to chlorine (*t*-test at 95% confidence).

Figure 2 compares the antimicrobial effect of nanoemulsified OEO treatment at different concentrations against a chlorine solution with 80 ppm, expressed as relative reductions (%) with respect to the chlorine treatment. While absolute reductions are expressed as log CFU/g, Figure 2 presents the same results normalized relative to the chlorine treatment (80 ppm), to facilitate comparison between treatments. For *S.* Enteritidis, the effect was concentration dependent, with the lowest concentration tested showing a microbial reduction (1.19 ± 0.55 log CFU/g) comparable to water, demonstrating the lack of antimicrobial effect. Increasing the OEO concentration resulted in higher effects, with the highest concentration tested having an antimicrobial effect comparable to the conventional 80 ppm chlorine solution (2.02 ± 0.43 log CFU/g). A similar trend was observed for the tomatoes inoculated with *E. coli*: the lowest concentration tested had practically no antibacterial effect, whereas the highest concentration again showed reductions (1.71 ± 0.36 log CFU/g) comparable to the conventional 80 ppm chlorine solution.

The concentration-dependent antimicrobial activity observed in EO nanoemulsions is likely related not only to the increased availability of bioactive compounds but also to concentration-induced changes in nanoemulsion behavior. Higher EO concentrations increase the amount of antimicrobial molecules available for bacterial interaction, while potentially affecting droplet dispersion, interfacial properties, and compound release (Yousef et al., 2019). The small droplet size of nanoemulsions enhances mass transfer and facilitates the delivery of active compounds to microbial cells, contributing to improved antimicrobial performance (Cecchini et al., 2021). Additionally, these results are aligned with previous studies from our group, showing that the antimicrobial efficacy of EO-based solutions is most probably influenced by EO concentration and the target microorganism. In the present study, increasing OEO concentration significantly improved bacterial reductions (*t*-test; *p* < 0.05), although a plateau was observed at higher concentrations. Similar concentration-dependent effects have been reported for other EO nanoemulsions, with Bodea et al. (2023) observing that higher concentrations of nanoemulsified oregano or rosemary EOs resulted in higher reductions of *E. coli*, and Luciano et al. (2025) reporting a similar trend for limonene against *Staphylococcus aureus* and *Candida guilliermondii* (previously *Meyerozyma guilliermondii*).

On the other hand, the nanoemulsified OEO treatment showed no significant effect against *L. monocytogenes* inoculated on cherry tomatoes. For every concentration tested, the microbial reduction attained (1.37 ± 0.15 log CFU/g) was comparable to that observed for water washing. This result is contrary to the commonly reported trend because, typically, Gram-negative bacteria are expected to show higher resistance to chemical agents than Gram-positive species. However, previous studies obtained similar results. Guerra-Rosas et al. (2017), observed that *E. coli* exhibited greater sensitivity to the antimicrobial action of mandarin EO based nanoemulsions than *L. innocua*. These findings highlight that the antimicrobial efficacy cannot be predicted solely based on bacterial classification, as the response depends on both the microorganism and the chemical composition of the EO. This variability is likely related to the complex composition of EOs, which can interact with multiple cellular targets (Bakkali et al., 2008). Citrus EOs, which are rich in lipophilic compounds such as D-limonene, can interact with bacterial membranes, increasing permeability and causing leakage of intracellular components, loss of membrane integrity, and ultimately cell death (Bakkali et al., 2008; Guerra-Rosas et al., 2017; Medeleanu et al., 2023). The incorporation of EOs in nanoemulsions may enhance the dispersion and delivery of hydrophobic compounds, such as D-limonene, improving their interaction with bacterial cells (Bhargava et al., 2015; Guerra-Rosas et al., 2017). However, antimicrobial efficacy may still vary depending on bacteria membrane composition, stress adaptation mechanisms, biofilm formation, strain variability, and the chemical composition and purity of the EO (Bakkali et al., 2008). *In vitro*, Gram-negative bacteria are generally more resistant to EOs than Gram-positive, because their lipopolysaccharide outer membrane limits the penetration of hydrophobic compounds (Bhargava et al., 2015; Calo et al., 2015; Angane et al., 2022). Additionally, higher concentrations of EOs are generally required to achieve comparable antibacterial effects in food matrices compared to *in vitro* conditions (Fisher and Phillips, 2006). Still, although *E. coli* and *S.* Enteritidis are both Gram-negative bacteria, their different responses to chlorine may be related to specific physiological characteristics, including differences in membrane properties and stress response mechanisms. Thus, chlorine susceptibility cannot be predicted solely by Gram classification, emphasizing the need to evaluate the direct effect of sanitizers against multiple foodborne pathogens.

Microscopy confirmed structural disruption, with the extent and type of damage varying between bacterial species even under the same EO treatment (Guerra-Rosas et al., 2017). Similar to our results, Guerra-Rosas et al. (2017) observed a higher antimicrobial activity of oregano, thyme, lemongrass or mandarin EO-pectin nanoemulsions against *E. coli* than *L. innocua*, regardless of EO type. TEM analysis showed severe damage in *E. coli* cells, including disruption of the cytoplasm and cytoplasmic membrane, leading to cell death. *L. innocua* showed higher resistance to EO-pectin nanoemulsions, likely due to its thicker outer membrane. TEM images confirmed that oregano and lemongrass nanoemulsions caused membrane pores and cytoplasmic damage in both bacteria, whereas thyme and mandarin induced less structural disruption.

The decontamination efficiency could potentially be enhanced while reducing the required EO concentration through the use of EO combinations. Synergistic interactions between different EOs have been previously reported to improve the antibacterial effectiveness compared to individual EOs (de Azeredo et al., 2011; Gurtler and Garner, 2022). Thus, future studies should aim at determining the maximum EO concentrations that ensure antibacterial efficacy without adversely affecting organoleptic properties enhancing overall effectiveness. The use of low EO concentrations may offer a safe and effective sanitization strategy for fresh produce, however, economic feasibility remains an important consideration. Although nanoemulsion EO formulations may involve higher production costs than conventional chlorine-based sanitizers due to additional processing requirements, their potential advantages, including improved dispersion, enhanced antimicrobial performance, and the valorization of agro-industrial by-products, may provide added value. Therefore, future work should also evaluate the cost–benefit balance of EO-based sanitization and preservation approaches.

#### 3.2.2 Effect of the contact time on the antimicrobial effect of the alternative wash

The results from the previous section showed that an OEO concentration greater than 10% would be required to attain antimicrobial effects for *E. coli* and *S.* Enteritidis similar to the conventional chlorine solution. Such high concentrations would have several disadvantages, increase the cost of the solution and potentially affect the sensory profile of the product. Thus, additional experiments were performed, to evaluate the effect of washing time using an OEO concentration of 10%. *L. monocytogenes* was excluded from further experiments, as the OEO solution proved ineffective even at the highest concentration.

**FIGURE 3:**
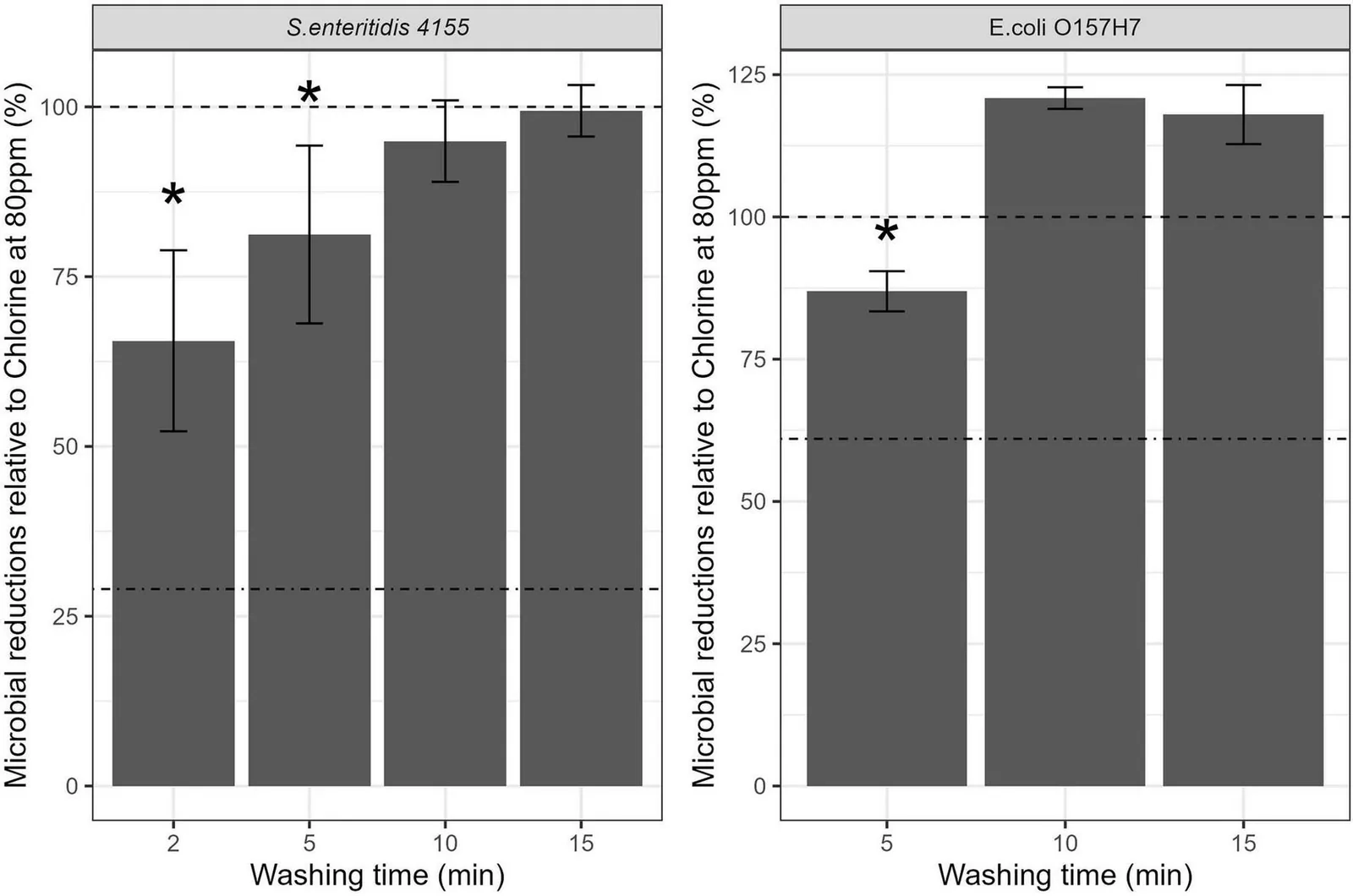
The relative bacterial reduction (% of log-reductions) obtained with nanoemulsified OEO (10%) and different washing times (2–15 min) against *Salmonella* Enteritidis and *E. coli* O157:H7 on cherry tomatoes. Values are expressed as percentages of the log reductions obtained with a chlorine treatment at 80 ppm (100%) (–). The dotted line represents the reductions obtained with water washing (.-.). Error bars indicate standard deviation. Asterisks (*) denote significant differences compared to chlorine (*t*-test at 95% confidence).

As illustrated in Figure 3, washing time had a significant effect on the antimicrobial activity of the OEO solution. The highest treatment time (15 min) resulted in reductions comparable to those obtained with the 80 ppm chlorine solution against both *E. coli and S.* Enteritidis demonstrating the effectiveness of this strategy under the tested conditions. These results suggest that nanoemulsified OEO shows potential as a chlorine-free alternative for produce washing under domestic conditions, where longer contact times are feasible.

These results agree with previous reports on the effect of the washing time on disinfection efficacy, as observed for nanoemulsified limonene against *S. aureus* and *M. guilliermondii* (Luciano et al., 2025) or for chlorine against *E. coli* O157:H7 and *Salmonella* (Wang et al. (2023). However, the relationship between the washing time and the bacterial reduction is not always linear, and excessive washing time may not yield proportional benefits, as shown by Lu and Wu (2010) who observed that there was no significant difference in the reduction of *Salmonella enterica* serovars when 5 and 10 min washing times were used. Our results align with that observation, showing a plateau in antimicrobial efficacy for longer times. The plateau observed at higher OEO concentrations may indicate that a maximum antimicrobial threshold was reached, beyond which additional EO loading provided limited improvements. This behavior may be associated with the saturation of EO interactions with bacterial membranes, where sufficient hydrophobic compounds are already available to disrupt membrane integrity and increase permeability (Chouhan et al., 2017; El-Sakhawy et al., 2026). Other proposed mechanisms, such as depletion of cellular energy reserves and inhibition of essential metabolic processes, may also contribute to antimicrobial activity (Falleh, 2025). However, these hypotheses were not directly evaluated in the present study. Therefore, determining the optimal washing time is crucial for balancing bacterial reduction with operational efficiency, particularly in the context of domestic washing practices, where time and convenience are key factors.

Despite the promising antimicrobial performance observed, some limitations of the present study should be acknowledged. The experiments were performed under controlled laboratory conditions, and further validation under real domestic or industrial washing scenarios is required to confirm the effectiveness and practical feasibility of OEO nanoemulsion-based washing solutions. A comprehensive quality evaluation, including the potential impact of residual OEO on tomato sensory properties, produce quality during storage, and consumer exposure, should be addressed in future studies, to further assess the practical applicability of OEO nanoemulsion-based washing solutions. Additionally, the economic feasibility of scaling up nanoemulsion production needs to be evaluated. Therefore, the results presented here should be considered preliminary proof-of-concept findings that provide a foundation for further investigation into the application of nanoemulsified EOs as alternative washing solutions for fresh produce.

### 3.3 Impact on consumer protection against salmonellosis when using a nanoemulsified OEO washing solution as an alternative to chlorine

The empirical results obtained showed that the washing solution based on nanoemulsified OEO could be an alternative to current solutions based on chlorine compounds for *Salmonella* decontamination. Therefore, a QMRA was developed to quantify its potential impact on consumer health. First, a predictive model was developed to describe the effect of changes in the washing time on the antimicrobial effect of the OEO solution, so these factors could be included into the QMRA calculations. The model was fitted by nonlinear regression, obtaining the model parameters reported in Table 1, which adequately described the observed antimicrobial effect (Supplementary Figure S1).

Parameter estimates are reasonable based on the data obtained. Parameter *a* represents a theoretical inactivation when the washing time is equal to zero. This parameter is close to the number of reductions obtained for the water washing (Figure 1), representing a sort of baseline. Parameter *b* shows the ideal maximum potential increase in log-reductions with respect to that baseline. The addition of both parameters yields 4.1 log-reductions (1.5 + 2.6), slightly higher than the maximum number of reductions observed experimentally (3.5 log CFU/g). This underlines the risks of using this model outside of the empirical range (Supplementary Figure S1).

The predictive models were used in combination with suitable probability distributions to simulate potential exposure scenarios for *Salmonella* linked to tomato consumption. This approach strengthens the practical relevance of the study by linking experimental reductions with their potential impact on consumer exposure and risk. As reported in Table 2, the use of a chlorine washing at 160 ppm significantly reduces the risk with respect to a water wash, decreasing the expected concentration at consumption in almost 2 log CFU/g. This causes a 20-fold reduction in the expected number of cases.

Current legislation, which includes a reduction in chlorine concentration of disinfectant solutions from 160 to 80 ppm, was predicted by the QMRA model to result in a slight increase in *S.* Enteritidis exposure from −0.98 to −0.81 log CFU/g. Accordingly, this change corresponded to a predicted 50% increase in the expected number of salmonellosis cases associated with tomato consumption.

According to the QMRA results, the alternative OEO washing solution would result in risk levels between the chlorine wash at 80 ppm and 160 ppm, presenting a 12% increase in the expected number of salmonellosis cases with respect to the 160 ppm scenario. Although this result is based on strong simplifications regarding the domestic washing time and storage conditions, those apply equally to all scenarios. Therefore, even if the risk estimates are not realistic, the relative comparison between them (i.e., that the EO solution would be more effective than the 80 ppm commercial solution, but less effective than the 160 ppm solution) would be most likely valid for more realistic conditions.

Another factor to consider is the potential presence of organic matter, as this may potentially reduce the antimicrobial effect of the EO. We considered for the assessment that household kitchens follow basic hygiene (such as using clean vases), so the organic matter would be lower than in industrial settings where several tons of produce are washed without water renovation. Nonetheless, the application of this alternative solution would require additional warnings, as it is likely to be more sensitive than chlorine-based solutions.

A final limitation of the QMRA is the use of predictive models based on a laboratory system. Actual washing solutions would introduce other factors (such as interaction between different vegetable matrices, agitation) that would impact the effectiveness of both the commercial and alternative washing solutions. This is typical source of uncertainty for QMRA models, where both microbial growth and inactivation experiments are based on limited data gathered under laboratory conditions (Messens et al., 2023). Therefore, the development of more realistic model systems that more realistically represent consumer behavior presents a future line of research.

## 4 Conclusion

Chlorine washing was effective against *E. coli*, *S.* Enteritidis, and *L. monocytogenes*, whereas water washing showed no significant antibacterial effect. The decontamination efficacy of the nanoemulsified OEO treatments varied depending on the bacterial species and OEO concentration. The antibacterial effect of the washing solutions increased linearly with increasing the concentration of nanoemulsified OEO. Overall, the highest concentration (10%) was effective against *E. coli* and *S*. Enteritidis, achieving reductions of up to 2 log CFU/g. However, it showed limited antibacterial activity against *L. monocytogenes*, with only about 1 log CFU/g reduction observed, and this effect was not significantly improved by increasing the concentration. The washing time had a significant influence on the effectiveness of the OEO solution. The 15 min treatment was effective against both *E. coli* and *S.* Enteritidis, reducing up to 2.5 and 3.5 log CFU/g respectively, demonstrating the effectiveness of this approach in conditions compatible with domestic washing practices. The QMRA indicated that the OEO-based washing solution results in risk levels between those associated with chlorine washes, with a 12% increase in the expected number of salmonellosis cases compared to the 160 ppm scenario. These results highlight the potential of nanoemulsified OEO as a promising chlorine-free alternative for the domestic washing of fresh produce, offering a more environmentally friendly approach aligned with the growing demand for sustainable and clean-label disinfection strategies. Still, these findings should be considered preliminary proof-of-concept obtained under controlled laboratory conditions, yielding results that provide a foundation for further investigation into the application of these washing solutions. Nevertheless, some limitations should be acknowledged, including the need for validation under realistic domestic and industrial washing scenarios, as well as further evaluation of produce quality, sensory properties, residual EO levels, consumer exposure, and economic feasibility. In the context of domestic washing treatments, produce key quality attributes should be systematically assessed to verify both consumer acceptability and practical value. In addition, future studies should explore the synergistic application of EOs, to determine whether combined treatments can further enhance microbial safety while preserving sensory and nutritional quality. Furthermore, the extrapolation of these results must be validated under real domestic conditions to confirm the effectiveness, feasibility, and consumer relevance of the treatment in practical scenarios.

## Data Availability

The datasets presented in this study can be found in online repositories. The names of the repository/repositories and accession number(s) can be found at: https://github.com/albgarre/tomato-washing-Limonene.
