# Supplementary material for: Nanoemulsified orange essential oil for decontamination of some foodborne pathogens on cherry tomatoes during simulated domestic washing
Source: Front Microbiol. 2026 Sep 7;17:1931295. doi: 10.3389/fmicb.2026.1931295 (PMC13590717; doi:10.3389/fmicb.2026.1931295)
Supplement: Supplementary file 1 [file Data_Sheet_1.pdf]

MASTERSIZER 2000

Result Analysis Report

**Sample Name:**<br>E00 - Average	**SOP Name:**	**Measured:**<br>miércoles, 16 de julio de 2025 12:35:01
**Sample Source & type:**	**Measured by:**<br>SAIT	**Analysed:**<br>miércoles, 16 de julio de 2025 12:35:03
**Sample bulk lot ref:**	**Result Source:**<br>Averaged	

**Particle Name:**<br>Limoneno	**Accessory Name:**<br>Hydro 2000SM (A)	**Analysis model:**<br>General purpose	**Sensitivity:**<br>Normal
**Particle RI:**<br>1.475	**Absorption:**<br>1	**Size range:**<br>0.020 to 2000.000 um	**Obscuration:**<br>4.83 %
**Dispersant Name:**<br>Dry dispersion	**Dispersant RI:**<br>1.000	**Weighted Residual:**<br>9.435 %	**Result Emulation:**<br>Off

**Concentration:**<br>0.0002 %Vol	**Span :**<br>7.952	**Uniformity:**<br>4.14	**Result units:**<br>Volume
**Specific Surface Area:**<br>27.5 m²/g	**Surface Weighted Mean D[3,2]:**<br>0.218 um	**Vol. Weighted Mean D[4,3]:**<br>0.989 um	

d(0.1): 0.136 um **d(0.5): 0.201 um** **d(0.9): 1.731 um**

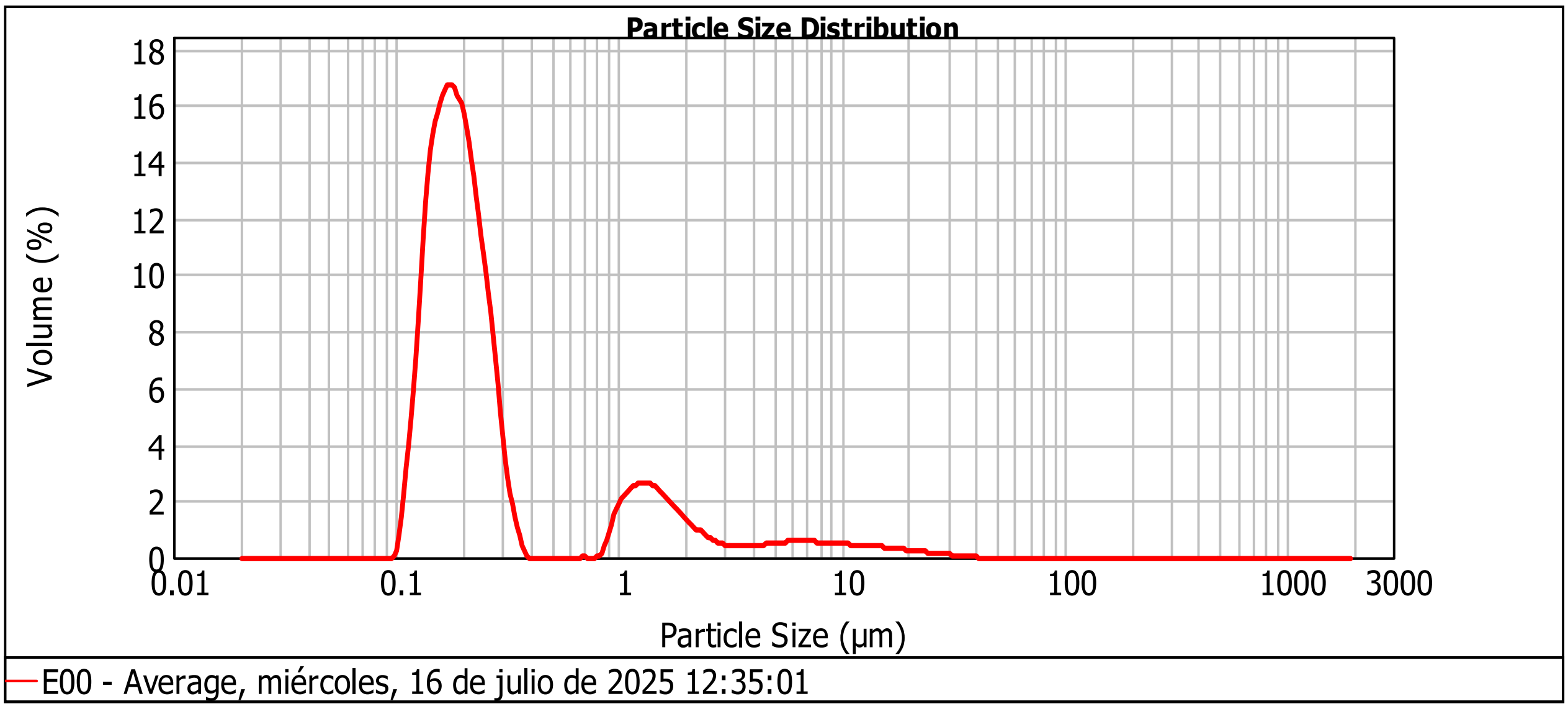

Size (µm)	Volume In %	Size (µm)	Volume In %	Size (µm)	Volume In %	Size (µm)	Volume In %	Size (µm)	Volume In %	Size (µm)	Volume In %
0.020	0.00	0.142	11.47	1.002	1.65	7.096	0.42	50.238	0.00	355.656	0.00
0.022	0.00	0.159	12.48	1.125	1.93	7.962	0.40	56.368	0.00	399.052	0.00
0.025	0.00	0.178	12.43	1.262	1.99	8.934	0.38	63.246	0.00	447.744	0.00
0.028	0.00	0.200	10.95	1.416	1.84	10.024	0.35	70.963	0.00	502.377	0.00
0.032	0.00	0.224	8.70	1.589	1.56	11.247	0.33	79.621	0.00	563.677	0.00
0.036	0.00	0.252	6.35	1.783	1.23	12.619	0.31	89.337	0.00	632.456	0.00
0.040	0.00	0.283	3.18	2.000	0.91	14.159	0.29	100.237	0.00	709.627	0.00
0.045	0.00	0.317	1.33	2.244	0.64	15.887	0.26	112.468	0.00	796.214	0.00
0.050	0.00	0.356	0.04	2.518	0.45	17.825	0.22	126.191	0.00	893.367	0.00
0.056	0.00	0.399	0.00	2.825	0.34	20.000	0.19	141.589	0.00	1002.374	0.00
0.063	0.00	0.448	0.00	3.170	0.30	22.440	0.15	158.866	0.00	1124.683	0.00
0.071	0.00	0.502	0.00	3.557	0.30	25.179	0.10	178.250	0.00	1261.915	0.00
0.080	0.00	0.564	0.00	3.991	0.33	28.251	0.07	200.000	0.00	1415.892	0.00
0.089	0.00	0.632	0.00	4.477	0.37	31.698	0.05	224.404	0.00	1588.656	0.00
0.100	0.83	0.710	0.00	5.024	0.40	35.566	0.02	251.785	0.00	1782.502	0.00
0.112	3.90	0.796	0.08	5.637	0.42	39.905	0.00	282.508	0.00	2000.000	
0.126	8.60	0.893	1.05	6.325	0.43	44.774	0.00	316.979	0.00		
0.142		1.002		7.096		50.238		355.656			

Operator notes:
