# Supplementary material for: Nanoemulsified orange essential oil for decontamination of some foodborne pathogens on cherry tomatoes during simulated domestic washing
Source: Front Microbiol. 2026 Sep 7;17:1931295. doi: 10.3389/fmicb.2026.1931295 (PMC13590717; doi:10.3389/fmicb.2026.1931295)
Supplement: Supplementary file 2 [file Supplementary_file_1.docx]

Supplementary Material

**Supplementary Figure 1.** Comparison between the microbial reductions of Salmonella enteritidis observed for different orange EO contact time, including the fitted model (--).
